# Smart Microneedles with Porous Polymer Layer for Glucose-Responsive Insulin Delivery

**DOI:** 10.3390/pharmaceutics12070606

**Published:** 2020-06-30

**Authors:** Asad Ullah, Hye Jin Choi, Mijin Jang, Sanghyun An, Gyu Man Kim

**Affiliations:** 1 School of Mechanical Engineering, Kyungpook National University, Daegu 41566, Korea; sasadullah84@gmail.com (A.U.); gbksj5@naver.com (H.J.C.); 2 Daegu Gyeongbuk Medical Innovation Foundation, Laboratory Animal Center, Daegu 41061, Korea; mijin22@dgmif.re.kr (M.J.); ash4235@dgmif.re.kr (S.A.)

**Keywords:** glucose-responsive, insulin delivery, porous coated-microneedles, sodium bicarbonate

## Abstract

A closed-loop system imitating the function of pancreatic cells, connected to microneedles (MNs) that automatically “release” insulin in response to the blood glucose (BG) levels would be highly satisfactory for improving the quality of life and health for diabetes patients. This paper describes an easy, fast and simple technique of coating a porous polymer layer on stainless steel (SS) MNs that release insulin in a glucose-responsive fashion. It was fabricated by sealing insulin, sodium bicarbonate (a pH-sensitive element [NaHCO_3_]) and glucose oxidase (glucose-specific enzymes [GOx]) into the pores of a porous polymer coating. Glucose can passively diffuse into the pores and become oxidized to gluconic acid by GOx, thereby causing a decrease in local pH. The subsequent reaction of protons with NaHCO_3_ forms carbon dioxide (CO_2_) which creates pressure inside the pores, thereby rupturing the thin polymer film and releasing the encapsulated insulin. Field emission scanning electron microscopy (FE-SEM) images displayed that upon the exposure of MNs to glucose-free phosphate buffer saline (PBS) with pH 7.4, the pores of the porous MNs were closed, while in MNs exposed to a hyperglycemic glucose level, the pores were opened and the thin film burst. These MNs demonstrated both in vitro (in porcine skin and PBS) and in vivo (in diabetic rats) glucose-mediated insulin release under hyperglycemic conditions with rapid responsiveness. This study validated that the release of insulin from porous MNs was effectively correlated with glucose concentration.

## 1. Introduction

Diabetes is one of the most universal persevering diseases, arising from a chronic metabolic disorder, in which glucose accumulates in the blood, causing a failure in the regulation of blood glucose levels [1]. Other complications may develop including retinopathy, cancer, cardiovascular disease, and chronic kidney disease [2]. The common treatment for diabetic patients is to maintain normal glucose levels in the blood by insulin injection [3]. Diabetic patients are required to take blood samples frequently which causes inconvenience and pain [4].

Microneedles (MNs), a promising noninvasive drug delivery system, have the ability of providing a painless self-administered alternative [5]. MNs can be transiently inserted into the stratum corneum to penetrate the epidermis and prevent injury to deeper tissue. When the outer skin layers are penetrated, the payload therapeutic formulation begins to diffuse [1,6]. MN drug delivery provides promising practical and clinical advantages compared with more traditional routes by enhancing therapeutic effectiveness through sustained and controlled release [7,8]. Clinical effectiveness of treatment through a more efficient administration of therapeutics and high molecular weight bioactive materials can be achieved using microneedles in a painless manner [9,10]. Biodegradable and biocompatible polymers are broadly used to make microneedles for the systemic drug delivery of vaccines, genes and chemotherapeutic agents [11,12]. These microneedles release their encapsulated payload over an extended time (several months) as a result of slow degradation and surface erosion of the polymer [13]. However, for many therapeutic applications, depending on the needs of the patient or physiological circumstances, it is often desirable to rapidly and selectively release the encapsulated drug formulations so as to provide a drug delivery in a controllable fashion [14,15]. The emergence of triggerable drug-delivery systems has attracted increasing interest as “smart” drug-delivery systems for overcoming the limitations of conventional drug delivery systems. That is, these systems can deliver drug formulations in a controlled fashion at a specific site and time, which results in high therapeutic efficiency [15,16,17,18]. Similarly, on-demand insulin delivery, desirable for diabetic patients, can be achieved with triggerable delivery systems. In this way, blood glucose levels are maintained and the risk of hypoglycemia, caused by the release of excess drug, is reduced [16,19]. Many pH [19,20] ultrasound [21] or light-responsive [22] polymer and hydrogel microcarriers [23] have been extensively explored for triggered drug release, but few of them can be easily prepared with simultaneous stimulus-sensitivity and biocompatibility [17,24,25]. Moreover, most smart drug delivery systems make use of several chemical and slow processes. First, through various chemical processes, the smart microcarriers are prepared which is followed by the administration to the body either by using hypodermic injection or combined with MNs [17,26,27,28]. Therefore, it is intuitive that a closed-loop system, connected to MNs that automatically “release” insulin in response to the levels of BG, with the biocompatibility and flexibility of polymer materials, and an efficient approach of preparation, reduces the risk related to conventional treatment of diabetic patients.

**Figure 1:**
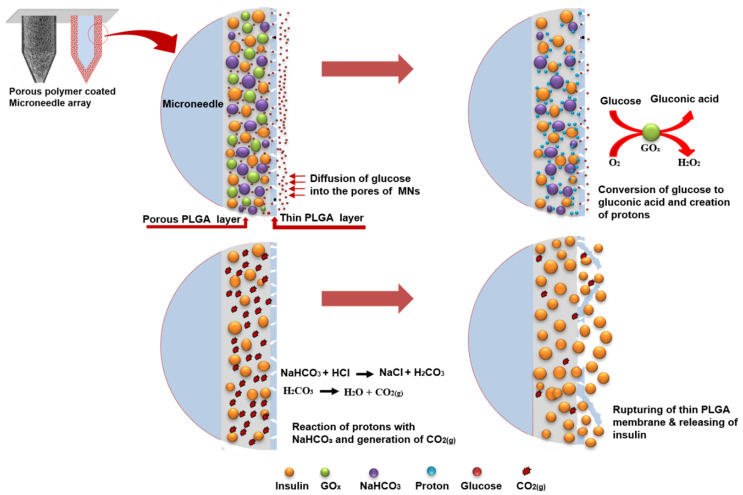
Schematic demonstration of the glucose-responsive insulin delivery system using porous coated microneedles (MNs).

This study demonstrates a very quick, simple and robust method of fabricating a glucose-responsive insulin delivery system based on MNs. We coated SS microneedles with a porous polymer layer that contained a glucose-responsive formulation for self-regulated insulin delivery in response to glucose concentration. A schematic illustration of this glucose-responsive release system and its operation is shown in Figure 1. Insulin, glucose oxidase (glucose-specific enzymes [GOx]) and sodium bicarbonate (pH-sensitive element [NaHCO_3_]) were encapsulated in the pores of the porous polymer layer by an emulsion method. The porous coatings were fabricated on MNs by dip-coating. The pores were capped by coating another thin poly(lactic-*co*-glycolic acid) (PLGA) film on the top of this porous layer to avoid the exposure of the glucose-responsive formulation while allow preloaded insulin to be released in response to specific glucose concentrations. Passive diffusion of glucose may occur across the thin PLGA film into the pores and oxidize into gluconic acid in the presence of GOx, consequently reducing the local pH [17]. In this acidic microenvironment, NaHCO_3_ decomposes to carbon dioxide (CO_2_) gas [26] which in turn generates pressure inside the pores causing them to rupture the thin polymer film and releasing the encapsulated insulin. Insulin release profiles were scrutinized at different glucose concentrations in vitro (in PBS and porcine skin) and in vivo in diabetic rats. Both the results obtained in vitro and in vivo revealed that the fabricated porous coated MNs were highly glucose responsive. In particular, the polymer coating was easy to prepare and required no particular expertise or technology, and simply required the dipping of MNs into the polymer solution to coat the solid microneedles.

## 2. Materials and Methods

### 2.1. Materials

These chemicals were procured from Sigma-Aldrich and used without additional purification: poly(vinyl alcohol) (98–99% hydrolyzed, MW: 31,000–50,000), polyethyleneimine (PEI, 50% [*w*/*v*] in water, analytical standard), dichloromethane (DCM, MW: 84.93 g/mol, anhydrous, ≥99.8%, mp: −97 °C, ρ: 1.325 g/mL), poly(lactic-*co*-glycolic acid) (PLGA, MW: 40,000–75,000, lactide: glycolide = 65:35), glucose oxidase (GOx), fluorescein isothiocyanate (FITC)-labeled insulin (insulin-FITC labeled human), insulin (human recombinant, insulin), hydrochloric acid (HCl), sodium hydroxide (NaOH), phosphate-buffered saline (PBS), streptozotocin (STZ), citric acid and sodium citrate dihydrate. U-BioMed Inc (Daegu, Korea) provided the stainless steel MNs, poly (methyl methacrylate) (PMMA) and sodium bicarbonate (NaHCO_3_) were obtained from a local market. All Sprague Dawley (SD) rats were acquired from Orient Bio Inc. (Seoul, Korea).

### 2.2. Fabrication of Porous-Coated MNs and Preparation of Coating Solution

Stainless steel MNs were kindly provided by U-BioMed, Inc. (Daegu, Korea) fabricated using micromachining technologies. A 7 × 7 array of microneedles was fabricated by inserting them into a plate of Poly (methyl methacrylate) of 10 mm square possessing a height of 4.5 mm. MNs possess height and diameter of 0.600 mm and 0.120 mm, respectively, on the array with intervals of 0.40 mm.

Using a water in oil emulsion technique, the polymer coating solution was prepared that was slightly modified from our previous work [29]. Briefly, 50 mg of NaHCO_3_ was dissolved in 1 mL of aqueous poly (vinyl alcohol) {(PVA) (10 mg/mL)} following the addition of glucose oxidase (GOx, 2 mg). By dissolving insulin in a hydrochloric acid (HCl) solution (0.01 M, pH = 2.0), an insulin solution (20 mg/mL) was prepared and afterwards the pH of the insulin solution was attuned to 7.0 using 1M sodium hydroxide (NaOH) solution. A total of 250 µL of the insulin solution (2% *w*/*v*) was added to the above (sodium bicarbonate) solution. The resulting (porogen) solution was emulsified with 4 mL of 2% (*w*/*v*) PLGA solution in DCM at 20,000 rpm for three minutes to prepare the water in oil emulsion (coating solution). Prior to plasma treatment, MNs were cleaned using ethanol, acetone and deionized (DI) water. After drying, the MNs were subjected to a discharge of oxygen plasma for five minutes by placing them in a plasma chamber. Discharge specifications of the plasma generator (RF-GEN, IDT Eng. Co., Carson, NV, USA) were as follows: frequency (13.56 MHz), time (5 min), oxygen pressure (0.2 mTorr) and plasma power (260 W). Then, MNs samples were taken out of the plasma chamber and put into an aqueous solution of PEI (20 mg/mL) for five minutes. After this, the samples were put into DI water for one minute three times and then dipped in the coating solution for five minutes to make the porous polymer coating on the MNs. This was followed by dipping MNs into another PLGA solution (5 mg/mL) to form a thin film on top of the porous coating. Finally, MNs samples were left at room temperature for two hours to allow the solvent to completely evaporate.

### 2.3. Characterization

The thickness and surface morphology of the glucose-responsive porous coatings were analyzed using FE-SEM (HITACHI S-4800, Tokyo, Japan) micrographs and Image-J software. All measurements were carried out in triplicate. After making the coating solution as explained in “Section 2.2”, the array of MNs was dipped in the coating solution for coating porous polymer layer and encapsulating insulin in the pores of porous coating on MNs. Drug loading efficiency was determined by measuring the concentrations of insulin in the coating solution before and after coating the MNs with UV spectrophotometry (Ocean Optics, USB 4000, Winter Park, FL, USA) [28]. The absorbance of a portion of the coating solution (1.5 mL) was measured before and after coating and from a standard curve of insulin, the quantity of insulin was computed. The drug loading efficiency (DLE) of insulin was computed using the formula given below:

(1) $$\begin{array}{l} \mathrm{Drug}\;\mathrm{loading}\;\mathrm{efficiency}\\ =\frac{\mathrm{quantity}\;\mathrm{of}\;\mathrm{insulin}\;\mathrm{before}\;\mathrm{coating}-\mathrm{quantity}\;\mathrm{of}\;\mathrm{insulin}\;\mathrm{remaining}\;\mathrm{after}\;\mathrm{coating}}{\;\mathrm{quantity}\;\mathrm{of}\;\mathrm{insulin}\;\mathrm{before}\;\mathrm{coating}}\times 100 \end{array}$$

In the same way we quantified the unloaded GOx in the coating solution before and after coating the MNs to measure the loading efficiency of it.

### 2.4. In Vitro Glucose-Responsive Insulin Release of Porous Coated MNs

In vitro insulin release from the developed MNs was investigated using PBS medium with different glucose concentrations at 37 °C. Four samples, each containing 49 MNs were put in vials containing 1.5 mL of PBS with different concentrations of glucose (0, 100, 200 and 400 mg/dL) in order to confirm the glucose-responsive capability of the porous coated MNs. Incubation of the vials were performed in a water bath at 37 °C. At pre-determined time points, samples were taken out for analysis. Released insulin content from porous coated MNs was determined using the UV absorbance along with a standard curve. By derivatizing the insulin’s known concentrations, a standard curve was found. After this, on the standard curve, the concentration of the released insulin was plotted, and the masses of the insulin released from MNs were computed. Five times, the experiments were repeated and for the analysis mean values were used. The quantity of insulin released in the medium was divided by the total quantity of insulin loaded for estimating the percent release of insulin.

### 2.5. Pulsatile Release and Parametric Study

With respect to the pulsatile release study, a sample of 49 MNs was first incubated in PBS (pH 7.4) with a glucose concentration of 100 mg/dL for 10 min. The sample was taken out and incubated in 400 mg/dL glucose solution for another 10 min at 37 °C. The cycle was repeated several times. The released insulin was measured by UV absorbance and a standard curve was established as described above. To determine the effect of NaHCO_3_ and glucose oxidase on insulin release, three samples were prepared. In the control sample {control (E = enzyme [GOx], B = backing soda [NaHCO_3_], I = insulin)}, the contents of NaHCO_3_, GOx and insulin were kept same to the entire study. In one sample, 1/2E (1/2E, B, I), the content of GOx was reduced to half, while in another sample, 1/2B (E, 1/2B, I), the NaHCO_3_ amount was reduced to half. Incubation of all three samples was performed in PBS (pH 7.4) with a glucose concentration of 100 mg/dL for 1 h at 37 °C. For the subsequent hour, samples were incubated in PBS (pH 7.4) with a glucose concentration of 200 mg/dL and finally were incubated in 400 mg/dL glucose solution at 37 °C for the same time. The cumulative release of insulin was computed using a standard curve and UV spectrometry.

### 2.6. Insulin Diffusion and Insertion Effectiveness of Porous-Coated MNs in Porcine Skin

Insulin diffusion and insertion effectiveness of these MNs was evaluated in porcine skin. FITC-labeled insulin was used as a model drug to be loaded into the porous coated MNs. The MNs containing FITC-labeled insulin, glucose oxidase and NaHCO_3_ were applied to porcine cadaver skin. From a local abattoir, fresh and full thickness pieces of porcine skin were sourced and stored immediately at −20 °C. Prior to the experiments, for one hour, the sourced skin pieces were defrosted at room temperature. With 75% alcohol, the skin surfaces were carefully cleaned and then air-dried for 10 min. After placing skin pieces on a fixed platform, the PBS containing glucose at a concentration of 400 mg/dL was inserted into the skin from the back side for controlling hyperglycemic level of glucose. MNs were then inserted into the skin manually at a hyperglycemic level (400 mg/dL) and controlled level (0 mg/dL) of glucose and both samples were put in an oven at 37 °C for five and thirty minutes. Afterwards, the samples were taken out from the oven and the MNs were removed.

### 2.7. Confocal Laser Scanning Microscope (CLSM)

After insulin delivery into porcine skin, for histological sectioning, drug delivered samples of appropriate sizes were cut and embedded within optimal cutting temperature (OCT) compounds. Using a microtome (RM2235, Leica, Seoul, Korea), the frozen OCT-skin tissues were sectioned into 30 μm thick slices. With the help of a confocal laser scanning microscope (CLSM; C2, Nikon Corporation, Seoul, Korea), the skin samples were analyzed for examining the release and diffusion of insulin from MNs after insertion. From the fluorescence images, MNs insertion depth and the insulin release could be directly observed.

### 2.8. In Vivo Glucose-Responsive Insulin Release in Diabetic Rats

The in vivo performance test for the developed MNs was conducted in accordance with the regulations for the care and use of laboratory animals, performed by the Experimental Animal Center of Daegu-Gyeongbuk Medical Innovation Foundation, Korea with the ethic committee approval number of “DGMIF-19052201-01” on 22 May, 2019. Sprague–Dawley (SD) rats (male, 200−250 g) were induced and used as a diabetic model by streptozotocin (STZ, sigma) according to previous reports [28,30]. Briefly, SD rats were fasted for 18 h, but drinking freely before a single intraperitoneal injection (IP) of a freshly prepared STZ solution at a dose of 65 mg/kg in citrate buffer (50 mM, pH 4.5). Blood glucose levels were measured seven days following STZ injection to ensure that they had reached a stable hyperglycemic level before the experiments. Blood samples were taken from the tail vein of the diabetic rats to measure glucose levels using a blood glucose meter (I-sens, Korea). SD rats showing a blood glucose level higher than 250 mg/dL were considered to be diabetic. The diabetic rats were divided into two groups randomly, with four rats in each group, and were fasted overnight prior to treatment. MNs loaded with insulin (10 mg/kg for each rat) along with (NaHCO_3_ + GOx), insulin-loaded microneedles, were applied to the rats transcutaneously in order to ensure that the drug delivery system had a controllable and glucose-responsive capacity. After application of the MNs, blood glucose levels for each diabetic rat were monitored for 10 h. Similarly, the blood glucose levels for the diabetic rats without MN application (control group) were also monitored for the same time period.

## 3. Results and Discussion

### 3.1. Fabrication of Glucose-Responsive MNs Array

For painless and convenient insulin administration in a glucose-responsive manner, SS MNs were coated with a uniform porous layer of PLGA which contained a glucose-responsive formulation for self-regulated delivery of insulin. They were fabricated by packing insulin, glucose oxidase and NaHCO_3_ into the pores of a porous polymer coating using a double emulsion process. A second, thin PLGA film was coated onto the porous layer to cap the drug and enzyme within the pores. The insulin loading content of porous-coated MNs was determined as 3.995 mg ± 0.1 and the loading efficiency was determined as 79.1 ± 0.5%.

**Figure 2:**
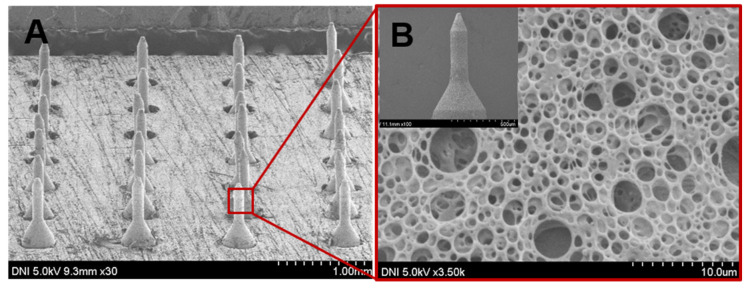
Scanning electron microscopy (SEM) micrographs of the glucose-responsive porous coated MNs-array.

The resulted MNs were arranged in a 7 × 7 array with intervals of 0.40 mm in a plate of Poly(methyl methacrylate) of 10 mm square as shown in Figure 2. We used PLGA, a biocompatible and biodegradable polymer, for the porous coatings on MNs. Here aqueous NaHCO_3_ was used as the porogen to incorporate pores into the polymer coating. NaHCO_3_ served two main functions. Firstly, it incorporated pores into the polymer layer. Secondly, it served as a stimulus-sensitive element in the porous polymer layer. The pore sizes and porosity of the porous coating were adaptable, and a desired porosity could be achieved by adjusting the aqueous sodium bicarbonate to polymer ratio in the coating solution, which in turn controlled the insulin content of the MNs. Increasing the aqueous NaHCO_3_ ratio to polymer solution in the emulsion and decreasing the homogenization rate increase the pore size and vice versa reported in our previous study [31,32]. Additionally, the porous coating thickness depends on the polymer concentration in the coating solution and the number of coated layers. Figure 2 displays SEM micrographs of the glucose-responsive MNs at different magnifications. All microneedles were smoothly coated with porous PLGA as shown in Figure 2A. The porous polymer coating was consistent, and the pores were uniformly distributed on the periphery of the MNs as shown in Figure 2B. Moreover, as the layer is composed of a polymer with a high molecular weight, mechanical stability of the porous layer is robust which can prevent premature loss of their payload. For one month and at 4 °C, no prominent morphological change or leakage of insulin was detected.

### 3.2. In Vitro Insulin Release of Porous Coated MNs in Response to Different Glucose Levels

**Figure 3:**
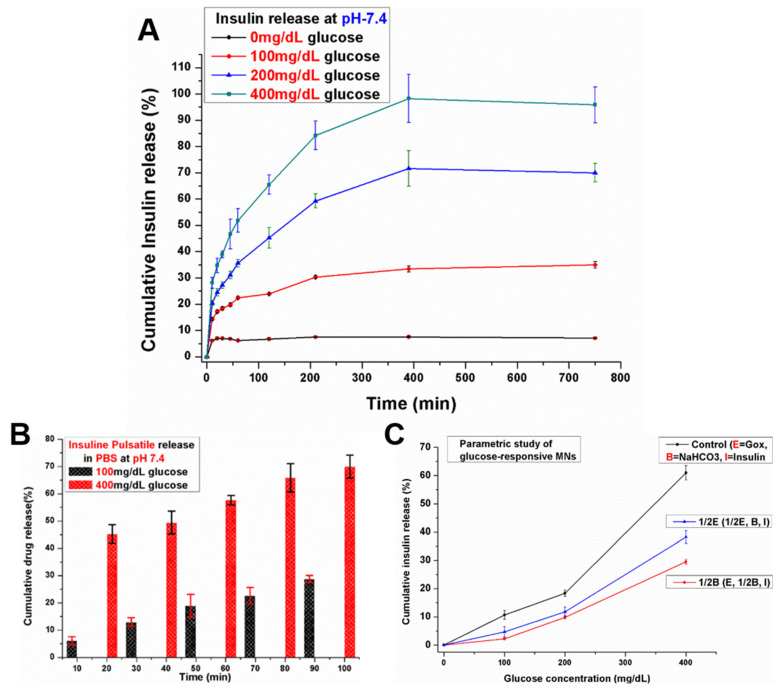
In vitro release profile of insulin for glucose-responsive porous polymer coated MNs incubated in phosphate buffer saline (PBS) with different glucose concentrations at 37 °C. (**A**) glucose-mediated insulin release at 0 mg/dL (black line), 100 mg/dL (red line), 200 mg/dL (blue line) and 400 mg/dL (green line); (**B**) Pulsatile release profile of insulin from porous coated MNs when the glucose concentration changed between 100 (black) and 400 (red) mg/dL alternatively for 10 min each; (**C**) The release rate (line slope) of insulin as a function of glucose concentration in the release media for {control (E, B, I)}, {1/2E (1/2E, B, I) (containing one-half amount of glucose-specific enzymes (GOx) compared to control (E, B, I))}, and {1/2B (E, 1/2B, I)}. Error bars indicate SD (*n* = 5).

Glucose can transport passively across the thin film of the PLGA into the pores of the porous layer. Using glucose oxidase, it is oxidized into gluconic acid, thereby causing the local pH to decrease. Sodium bicarbonate (NaHCO_3_) plays a key role in this system and it is incorporated easily into the pores of the porous polymer layer along with glucose oxidase and insulin. In this acidic microenvironment, NaHCO_3_ reacted with the protons to quickly generate CO_2_ bubbles. The generation of CO_2_ bubbles produced pressure inside the pores which created pores within the thin PLGA film or caused it to burst and thereby swiftly releasing insulin in a glucose-responsive fashion. As a result, by incorporating NaHCO_3_ and glucose oxidase in the aqueous core, we obtained a class of smart MNs that could unload promptly, in a glucose-mediated manner, the encapsulated drug. For glucose-responsive insulin-delivery applications, the effectiveness of the porous polymer-coated MNs was investigated by inspecting the in vitro insulin release profile from the glucose-responsive porous-coated MNs in response to different glucose levels. The porous coated MNs were incubated in PBS with different glucose levels (0, 100, 200, 400 mg/dL) at 37 °C. The medium was collected at given time intervals for analysis. As shown in Figure 3A, there was a negligible release of insulin from the porous coated MNs when exposed to a control solution of PBS with no glucose (0 mg/dL) for 12.5 h. Approximately 6% insulin release was observed in controls (0 mg/dL) within the first 1 h, while for the next 11.5 h incubation, no increase in the release of encapsulated insulin was observed. This suggested that there was no conversion of glucose to gluconic acid to reduce the local pH and consequently no reaction of acid (proton) with sodium bicarbonate to produce pressure because of the creation of carbon dioxide (CO_2_) inside the pores. Therefore, at normal physiological conditions with no glucose, the amount of insulin released from the porous polymer coated MNs was almost negligible. The small release of insulin at the initial 10 min may be due to residual insulin attached on the surface of the MNs and not from the portion packed inside the pores. No increase in insulin release was observed for the subsequent 12 h incubation. When the glucose-responsive porous coated MNs were exposed to normoglycemic levels (100 mg/dL), the amount of released insulin from porous coated MNs over 12.5 h was small. In insulin-dependent therapy, such a basal release rate of insulin at normoglycemic level is also desirable for managing fluctuations of blood-glucose. However, upon increasing the glucose concentration to 200 mg/dL in PBS, insulin release was increased correspondingly, and a profile of significantly higher insulin release was achieved for the sample exposed to the hyperglycemic level (400 mg/dL). Almost 53% of the encapsulated insulin was released within the first 1 h, while almost all the encapsulated insulin was released during the subsequent 5 h incubation.

The insulin release in Figure 3A is directly related to the concentration of glucose in the receiving medium, which is regulated by the pH stimulus of glucose metabolism. The proton generation by the conversion of glucose to gluconic acid catalyzed by glucose oxidase at normoglycemic level is relatively low, resulting in a relatively weaker reaction with NaHCO_3_. Consequently, this effect generated less pressure inside the pores, resulting in low insulin release at 100 mg/dL. However, a 400 mg/dL glucose solution accumulated more protons and decreased the pH value of the local environment, which was acidic enough to generate more pressure inside the pores and burst the thin PLGA film completely or created large pores resulting in a fast rate of insulin release. This confirmed that the release of insulin from the glucose-responsive MNs containing NaHCO_3_ and glucose-specific enzymes in the pores was dependent on glucose concentration. As mentioned earlier, in the presence of high glucose concentrations, more proton (H+) ions were created due to the conversion of glucose to gluconic acid. In this high acidic condition, protons (H+) reacted with sodium bicarbonate and led to the generation of CO_2_ bubbles inside the pores of MNs which caused more pressure. As a result, pores were created in the thin PLGA film or caused it to rupture, thus releasing the encapsulated insulin. More importantly, most of the pores were opened leading to the release of encapsulated insulin after the thin PLGA film ruptured initially. However, during the initial rupture of the thin PLGA film, some pores may still have not been totally exposed to the medium, so it took comparatively longer time to release the encapsulated insulin. Moreover, we observed a typical pulsatile pattern of insulin release after exposing the glucose-responsive porous coated MNs alternatively between hyperglycemic and normoglycemic solutions every 10 min for several cycles (Figure 3B). The release rates responded to the glucose level change in the incubation solution. We recorded a maximum of seven-fold difference in insulin release rate after switching the glucose levels.

Moreover, when the GOx content in the porous coated MNs was reduced by 50% {1/2E (1/2E, B, I)} vs {control (E, B, I)} (Figure 3C), the release rate of insulin concomitantly decreased. Similarly, when the quantity of NaHCO_3_ was decreased to half {1/2B (E, 1/2B, I)} vs {control (E, B, I)} in the porous coating, the rate of insulin release decreased more than that which was reduced using half the content of GOx as shown in Figure 3C. The results revealed that the quantity of NaHCO_3_ has a greater effect on the insulin release rate than GOx in the glucose-responsive porous coated MNs. This indicates that the insulin release rate can be regulated by changing the quantity of GOx and NaHCO_3_ in the developed MNs.

Collectively, the results affirmed that, from the porous coated MNs, the release of insulin was a glucose-dependent process. The observed concentration of insulin revealed the glucose-responsive release behavior of the developed porous coated MNs. In relation to the triggering of the microneedles drug delivery, this glucose-responsive release behavior is of interest.

**Figure 4:**
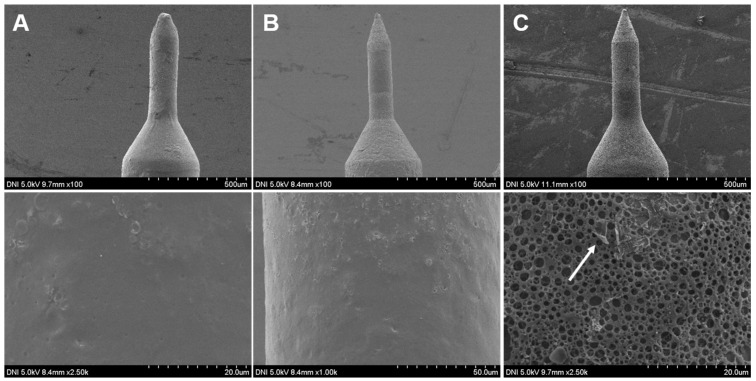
SEM micrographs of glucose-responsive porous coated MNs at different magnifications. (**A**) before incubation in PBS at pH 7.4 and 37 °C; (**B**) after incubation in PBS with 0 mg/dL glucose concentration at pH 7.4 and 37 °C; (**C**) after incubation in PBS with 400 mg/dL glucose concentration at pH 7.4 and 37 °C. The morphology of the MNs before and after incubation at 0 mg/dL was the same. The pores were unexposed and thin poly(lactic-*co*-glycolic acid) (PLGA) film stayed intact, while the pores of the MNs incubated in PBS with 400 mg/dL at pH 7.4 were open and the thin PLGA film was ruptured.

Our hypothesis was further confirmed by obtaining FE-SEM images of the glucose-sensitive MNs before and after incubation under control and hyperglycemic conditions. Upon exposing the MNs to PBS without (0 mg/dL) glucose, the surface design of MNs was the same before and after the incubation. The pores were closed and the thin PLGA film stayed intact as shown in Figure 4A and 4B. On the contrary, the pores were open and the thin PLGA film was ruptured after incubating in PBS with hyperglycemic levels (400 md/dL) as shown in Figure 4C. Some traces of the burst thin polymer film were observed on the porous layer after incubating at the hyperglycemic level as indicated by the arrow in Figure 4C.

### 3.3. Insulin Diffusion and Insertion Effectiveness of Porous-Coated MNs in Porcine Skin

**Figure 5:**
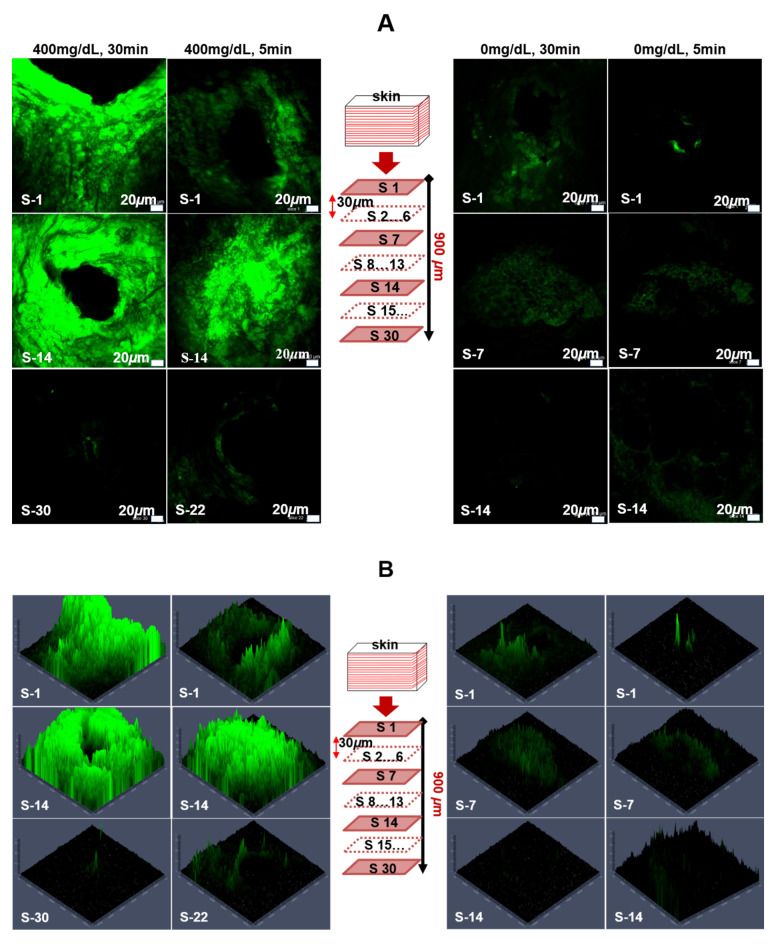
Confocal laser scanning micrographs of histological sections for fluorescein isothiocyanate (FITC)-labeled insulin delivery in porcine skin at 37 °C. Histological sections were obtained by slicing the FITC-labeled insulin delivered skin tissues using a microtome. A: (left side); FITC-labeled insulin delivered to skin at 400 mg/dL for 5 and 30 min, S-1 represents the top skin section, whereas S-30 represents the deepest section for which FITC-labeled insulin was detected. The thickness of each section was 30 μm. (**A**) (right side); FITC-labeled insulin delivered to skin at 0 mg/dL for 5- and 30-min. S-1 represents the top skin section, whereas S-14 represents the deepest section for which FITC-labeled insulin was detected. (**B**) Corresponding fluorescence intensities of FITC-labeled insulin delivered to skin at 400 mg/dL for 5 and 30 min (left side) and 0 mg/dL for 5 and 30 min (right side).

To investigate the skin penetration capability and painless delivery property of the glucose-sensitive porous coated microneedles, in vitro skin insertion tests were performed using the full-thickness porcine skin. MNs that contained FITC-labeled insulin were injected into porcine cadaver skin at 37 °C and their diffusion was monitored. In previous studies, it has been mentioned that in vivo delivery efficiency in mice is similar to that in vitro in porcine skin [33,34]. Figure 5A shows the CLSM images of the separated skin treated with FITC-labeled insulin-loaded MNs at control (0 mg/dL glucose) and hyperglycemic (400 mg/dL glucose) levels for 5 and 30 min. The results revealed that the encapsulated FITC-labeled insulin was successfully released in the skin in the hyperglycemic level. Fluorescence was observed in the deeper tissues. Significant delivery of the insulin occurred within 5 min. The strong green fluorescence at the surrounding area of the skin puncture sites revealed the diffusion of FITC-labeled insulin in the skin. To further investigate the distribution of drug in deeper tissues, confocal laser scanning microscopy images were acquired at various depths of the skin. The diffusion of FITC-labeled insulin was observed at a depth of more than 700 µm as shown in Figure 5A (left side). S-1 represents the top skin section following FITC delivery, whereas S-22 and S-30 represent the lowest skin sections at which fluorescence was observed by CLSM images for 5 and 30 min respectively, on the left side of Figure 5A. The thickness of each section was 30 μm. After inserting for 30 min, a strong green fluorescence at the surrounding area of the skin puncture site was evident, as shown in Figure 5A. In addition, the green fluorescence was observed in deeper tissues and on the large space of the surrounding area of the skin puncture sites, indicating diffusion and permeation of more insulin from the porous coated MNs.

In contrast, for skin at control levels, most of the FITC-labeled insulin remained within the pores of the MNs after insertion for 5 and 30 min. The weak green fluorescence was found around the area of the skin insert sites compared with that under hyperglycemic conditions. Almost no green fluorescence was observed in deeper tissues. Even after a 30 min application, very weak fluorescence was observed to a depth of 420 µm in the skin tissue, indicating that only a small amount of FITC-labeled insulin had diffused into the tissues as shown in the right side of Figure 5A. This small release of FITC-labeled insulin at control glucose levels may be attributed to FITC-labeled insulin that initially remained on the surface of the MNs at the time of coating or may be due to a minor release through the nanopores of the thin PLGA film.

We also observed the intensity of the fluorescence and diffusion areas covered by the released FITC-labeled insulin at various skin depths based on the obtained CLSM images. As revealed by the results displayed in Figure 5B, fluorescence intensity of the FITC-labeled insulin and the diffusion areas at hyperglycemic levels of glucose were significantly greater than the controls. A large amount of FITC-labeled insulin was observed under the puncture sites in the deeper tissues for the hyperglycemic level, even after 5 min of application and further increased when porous coated MNs were applied for 30 min as shown on the left side of Figure 5B. For the control level, a very weak intensity of fluorescence was observed which suggested that only a small amount of FITC-labeled insulin had been diffused into the tissues for 30 min of application (as shown on the right side of Figure 5B). It is believed that the high glucose level in the tissue can be triggered by the rapid release of insulin from the porous coated MNs. Both the results acquired in vitro in PBS and in porcine skin were commensurate and consistent with the previous observations. This revealed that our proposed porous polymer coated MNs show great promise to be used as “smart” glucose-responsive drug delivery systems.

### 3.4. In Vivo Glucose-Responsive Insulin Release in Diabetic Rats

Streptozotocin (STZ) is a widely used chemical for inducing diabetes in animal models, especially rodents (i.e., mice and rats), because it offers a simple, cost effective and readily available method [35,36,37]. Type 1 diabetes, which can be induced in rodents by a single intraperitoneal injection (IP) of STZ, causes insufficient endogenous insulin production and hyperglycemia due to the destruction of the pancreatic beta cells [38]. IP injection of STZ caused physiologic changes consistent with reports of chemically-induced diabetes in several species [39,40]. Furthermore, it has been reported that male pancreatic beta cells exhibit more STZ-induced cytotoxicity than that of females [41]. Therefore, in this study, diabetes was induced by a single, high-dose STZ injection (65 mg/kg) into male SD rats.

**Figure 6:**
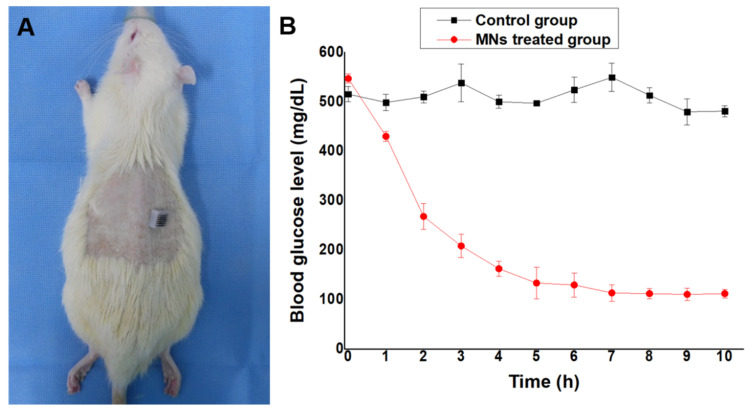
In vivo insulin release in diabetic rats. (**A**) Photograph of diabetic rat treated with MNs. (**B**) blood glucose levels after application of control and insulin loaded MNs. Error bars indicate SD (*n* = 4).

As shown in Figure 6A, the diabetic SD rat induced by STZ was treated by the glucose-responsive MNs-array. All diabetic rats were anesthetized with isoflurane (2~3%) and dorsally shaved before MNs-array treatment of the naked area. To investigate the performance of glucose-triggered MNs in vivo, we applied the fabricated MNs to diabetic rats (the blood glucose levels were 550 ± 10.5 mg/dL). Figure 6B shows the blood glucose levels (BGLs) of diabetic SD rats treated with insulin-loaded MNs and the control group (rats without applied MNs) after transdermal administration. In case of the insulin-loaded MNs group, the BGLs of diabetic SD rats exhibited a faster reduction rate in the first two hours and then decreased slowly for the next three hours until reaching normal glucose levels (100 ± 20 mg/dL). BGLs reached normal glucose levels after five hours of treatment and maintained similar levels for the following five hours (100 ± 20 mg/dL). However, the control group showed an insignificant decrease in BGLs for diabetic SD rats. The BGLs remained at the hyperglycemic level for the whole period.

To evaluate the biocompatibility of the system, the cytotoxicity of MNs was investigated. According to ISO-10993 guidelines, skin irritation (erythema, edema) is observed clinically after applying and removing the material for at least four hours. In this study, we applied the microneedle for about 10 h, removed it, and then clinically observed the dorsal skin of rat for three days. As a result, there was no erythema or edema and significant weight loss was not observed. This means that the microneedle is considered biocompatible and non-toxic.

The animal study also revealed that insulin release by the porous polymer coated MNs is directly related to the concentration of glucose, which is controlled by the pH stimulus of glucose metabolism. At high glucose concentrations, proton generation by the conversion of glucose to gluconic acid catalyzed by glucose oxidase was high, resulted in the fast rate of insulin release within the first two hours. However, when the glucose level was reduced to a normoglycemic range (<200 mg/dL), the insulin release rate was also decreased as discussed in the previous sections in detail. This confirmed that the release of insulin from the MNs containing NaHCO_3_ and glucose-specific enzymes in the pores was dependent on glucose concentration.

## 4. Conclusions

To summarize, using a glucose-responsive formulation in porous polymer coated MNs, we were able to develop a new insulin delivery system. Sodium bicarbonate, a pH-responsive component, was packed together with glucose-specific enzymes (GOx) and insulin in the pores of porous polymer coatings on SS MNs with a very simple and quick approach. This formulation demonstrated glucose-mediated insulin release under hyperglycemic conditions with rapid responsiveness both in vitro and in vivo. Importantly, the release rate of insulin was also reduced when the glucose levels reached the normoglycemic level, which could avoid the risk of hypoglycemia. As such, the proposed MNs could be used for the controlled release of insulin in a glucose-responsive fashion. Moreover, this system also provides the opportunity for the delivery of different therapeutic drugs to treat other diseases.
